# “When you get the HPV vaccine, it will prevent cervical cancer; it will act as a shield”: adolescent girls’ knowledge and perceptions regarding the human papillomavirus vaccine in Zambia

**DOI:** 10.3389/frhs.2023.1208458

**Published:** 2023-09-13

**Authors:** Mwansa Ketty Lubeya, Carla J. Chibwesha, Mulindi Mwanahamuntu, Moses Mukosha, Simone Frank, Mary Kawonga

**Affiliations:** ^1^ Department of Obstetrics and Gynaecology, School of Medicine, The University of Zambia, Lusaka, Zambia; ^2^ Women and Newborn Hospital, University Teaching Hospitals, Lusaka, Zambia; ^3^ School of Public Health, Faculty of Health Sciences, University of the Witwatersrand, Johannesburg, South Africa; ^4^ Clinical HIV Research Unit, Helen Joseph Hospital, Johannesburg, South Africa; ^5^ Department of Pharmacy, School of Health Sciences, University of Zambia, Lusaka, Zambia; ^6^ School of Medicine, North Carolina Translational and Clinical Sciences (NC TraCS) Institute, University of North Carolina at Chapel Hill, Chapel Hill, NC, United States; ^7^ Department of Public Health Medicine, Charlotte Maxeke Johannesburg Academic Hospital, Johannesburg, South Africa

**Keywords:** HPV vaccine acceptability, HPV vaccine uptake, COVID-19 vaccine, health belief model, parental consent, myths and misinformation, cervical cancer

## Abstract

**Introduction:**

The human papillomavirus (HPV) vaccination is an important preventive measure for HPV-related conditions such as cervical cancer. In 2019, Zambia introduced a free national HPV vaccination program for 14-year-old girls. However, the adolescents’ knowledge and perceptions regarding the HPV vaccine are not well understood. Therefore, this study aimed to understand adolescent girls’ knowledge and perceptions regarding the HPV vaccine and discuss its acceptability and uptake implications.

**Methods:**

We conducted a qualitative study in the Lusaka district between June 2021 and November 2021 using semi-structured interviews with adolescent girls aged 15–18 years regardless of their HPV vaccination status. Interviews were transcribed verbatim, and NVIVO 12 was used for data management and analysis. We coded transcripts deductively and inductively based on emerging themes. Perceptions were coded using the health belief model constructs.

**Results:**

We interviewed 30 adolescent girls to reach saturation. Seventeen girls reported having received at least one dose of the HPV vaccine. Participants expressed variable knowledge and awareness about HPV and the HPV vaccine. Participants exhibited positive attitudes towards the HPV vaccine and perceived it as beneficial. However, there were multiple perceived barriers to vaccination, such as the need for parental consent, not being in school, concerns about vaccine side effects, and belief in myths and misinformation

**Conclusion:**

The adolescent girls in this study showed variable knowledge and positive attitudes toward the HPV vaccine despite the many perceived barriers. To support increased HPV vaccine acceptability and uptake among adolescent girls in Zambia, it is critical to actively engage stakeholders involved in HPV vaccination, such as adolescents and their parents, and debunk myths and misconceptions about HPV vaccination. Health education in schools and communities should be implemented to increase knowledge about HPV and HPV vaccination among adolescents and their parents.

## Background

The World Health Organization (WHO) recommends that the human papillomavirus (HPV) vaccine be included in national vaccination programs to prevent cervical cancer and other HPV-related conditions (1). Persistent infection with HPV causes benign conditions such as genital warts and malignancies such as cervical cancer, anogenital cancers (anal, vulva, penile), and head and neck cancers. HPV is a risk factor for the development of cervical cancer, with types 16 and 18 commonly identified in approximately 70% of all cervical cancers (2). By far, cervical cancer is most common in Low and Middle-Income Countries (LMICs), with a disproportionately larger burden in sub-Saharan Africa (SSA) (1). In 2020, there were over 600,000 new cases of cervical cancer globally, with approximately 330,000 related deaths, most of which were reported from LMICs (1).

Like many other SSA countries, Zambia has one of the highest cervical cancer incidences and mortality rates in the world (2) and faces many challenges in treating those diagnosed with the disease since most of them present in late stages (3). According to the Global Cancer Observatory, Zambia cervical cancer-related incidences and mortality stand at 65.5 and 43.4 per 100,000 (1) making it one of the most common cancers in the country.

Persistent infection with HPV and rapid progression to malignancy is characteristic in women co-infected with the Human Immunodeficiency Virus (HIV), especially in high epidemic regions like Zambia (4–6). Women living with HIV are six times more likely to have cervical cancer than those without HIV with about 63.8% of women with cervical cancer and HIV residing in Southern Africa (7). The 2021 Zambia Population-based HIV/AIDS Impact Assessment (ZAMPHIA) reported a National HIV prevalence of 11% among people aged 15 years and older, however, when disaggregated by sex, women bear a higher prevalence of 13.9% while it is 8% in males (8). This further puts women at an increased risk of having persistent infection with HPV leading to cervical cancer.

In 2018, the WHO recognized cervical cancer as a public health problem and announced a strategy for its elimination by setting triple targets to be achieved by 2030 (9). According to this strategy, the first target entails that 90% of girls should be fully vaccinated against HPV by age 15 years and this is the focus of this paper. There is overwhelming evidence of the safety and effectiveness of the HPV vaccine as a primary prevention for cervical cancer (10).

The HPV vaccine is primarily recommended for adolescents aged 9–14 years (11), however, most countries in SSA are currently implementing girls-only programs (12). In contrast, high-income countries (HIC) have commenced gender-neutral programs for both girls and boys as the HPV vaccine is more accessible (11). Countries that have implemented national HPV vaccination programs, deliver vaccinations in various settings such as schools, health facilities, community outreach posts, or a combination of different platforms (13, 14).

Since 2019, Zambia has been offering the two-dose HPV vaccine to 14-year-old girls 12 months apart, following a demonstration project done between 2013 and 2017 in Lusaka Province (15). The national HPV vaccination program is campaign-based, conducted annually during the first round of Child Health Week (CHWk1), lasting six days from Monday to Saturday, after which walk-in vaccinations are at the health facilities for those who could have missed out during the campaign. The primary vaccination platforms include static at health facilities and outreach at schools and other community points. However, uptake has been low owing to different factors such as parental refusal, beliefs in myths and misinformation, school closures due to the COVID-19 pandemic and its prevention measures (15). Zambia's reported coverage for dose one in 2019 was 75%, which dropped to 39% in 2021 owing to some of these highlighted factors (16, 17).

Understanding adolescents’ knowledge and perceptions of the HPV vaccine is critical. Despite adolescents being the primary recipients (18), they are frequently left out in decision-making and research. In Zambia, most research has focused on other stakeholders, such as parents (6, 17, 19–22) and healthcare workers (22, 23) with very few studies focusing on adolescent girls as participants (24), despite being important stakeholders. The available literature has scanty information on adolescent girls’ knowledge and perceptions regarding the HPV vaccine and this is likely to impact acceptability and uptake (25).

Therefore, adolescents’ attitudes regarding the vaccine should be explored as a first step, to ensure the success of the HPV vaccination program as they are key stakeholders. This study is partly framed within the health belief model (HBM), a well-known and frequently used theory commonly used for exploring perceptions that may influence health-seeking behaviour such as HPV vaccine acceptability and uptake (26–29).

## Methods

### Study design and setting

We conducted an exploratory qualitative study using semi-structured interviews with adolescent girls between June 2021 and November 2021 within selected schools and health facilities of Lusaka district, the capital city of Zambia. The primary site for HPV vaccination is the school, as most adolescents within the target age group are found in schools. The Ministries of Health and General Education collaborate in implementing the HPV vaccination program. The number of eligible girls is determined using school registers, headcount, and Central Statistics Office figures (Now Zambia Statistics Office). At the same time, community health workers identify out-of-school girls in a door-to-door fashion or through civil societies and these girls receive the vaccine from health facilities or community outreach points.

### Sampling

In the first stage, schools enrolling adolescent girls aged 15–18 years within the subdistricts of Lusaka were sampled purposively as those participating in the HPV vaccination program. Thereafter, girls aged 15–18 years were selected based on their eligibility to receive the HPV vaccine, regardless of their vaccination status. The selection of this age group was based on Zambia's national HPV vaccination policy rolled out in 2019, which currently targets 14-year-old girls for dose one (30), which was preceded by a demonstration project between 2013 and 2017 targeting 9- and 10-year-old girls out of school or school grade 4 pupils (31). The included schools were sampled conveniently with a combination of public, community, and private schools. The communities served by the various schools are primarily urban either in low, medium, or high-density areas.

Eleven schools were included with a minimum of two eligible girls per school selected to participate in the study. Although participants in our study were not asked about their socioeconomic status, a recent study within the same setting which focused on parents showed that most participants were either in the middle or lower wealth quintiles (17). The researchers met the headteachers to get access to the schools to sample eligible girls. Adolescent girls were identified either through the class teacher or the focal point teacher for school health-related activities. The potentially eligible girls were met during class break times slated at different times of the day.

Out-of-school girls were identified with the help of community health volunteers. Information about the study was given to the girls, and if they were willing to participate, information sheets and consent forms were presented in English or Local language in a sealed envelope for the girls to deliver to their parents for consent purposes. The girls were asked to return the forms once the parents reviewed them, and those who agreed to participate signed the informed consent form. Assent to participate in the study and interview audio recordings were obtained from the adolescent girls after parental consent.

### Data collection

The semi-structured interview guide consisted of questions and probes that focused on socio-demographics; knowledge of HPV and the HPV vaccine, perceptions regarding the HPV vaccine, vaccination status (whether they had received at least one dose of the HPV vaccine), willingness to vaccinate for those not vaccinated or motivation for those vaccinated, and suggestions related to increasing HPV vaccine uptake. We explored knowledge and awareness by asking participants what they knew about HPV, what type of conditions it causes, if they knew about cervical cancer or genital warts and how they can be prevented, and how to prevent transmission. The questions regarding the HPV vaccine were whether they have heard about it, when it is given and how, and its purpose. To assess perceptions regarding the HPV vaccine, we used the HBM.

The HBM was developed in the early 1950s as a cognitive theory to predict health-seeking behavior (32). The original HBM postulates that for an individual to take a health-related action, they should perceive the disease as severe, perceive their susceptibility to the disease, believe that it is beneficial to take preventative action (HPV vaccination, in this case), and not perceive significant barriers to taking the health action (32). Two other constructs were added; self-efficacy (one's own confidence to engage in the behavior) and cues to action (specific triggers to action). Cues to action may be internal such as experiencing symptoms, or external such as receiving advice from friends, family members, or health care providers (32).

Some of the questions asked to the participants based on the HBM included; how likely are you to get infected with HPV, how severe would the HPV infection be if you got infected, what do you think are the benefits of getting the HPV vaccine, and what are the barriers to getting the HPV vaccine. See Table 1 for details.

**Table 1:** Examples of questions asked under each of the health belief model constructs.

HBM construct	Example question
Perceived susceptibility to HPV infection	How likely do you think are you to get infected with HPV?
Perceived severity of HPV infection	How severe would the HPV infection be if you got infected?
Perceived benefits of HPV vaccination	What do you think are the benefits of getting the HPV vaccine?
Perceived barriers to HPV vaccination	What are the possible things that can prevent you from receiving the HPV vaccine?
Cues to receive the HPV vaccine	What motivated you to get the HPV vaccine or why do you intend to get the HPV vaccine? (Depending on vaccination status or willingness to vaccinate)
Self-efficacy on getting the HPV vaccine	Could you please tell me more about your belief in your ability to receive the HPV vaccine?

An iterative process was used to develop and refine the interview guide. The guide was initially piloted using two interviews, after which edits were made to increase comprehension of the questions and improve clarity. Interviews took place in a private room on school premises for school girls and within health facilities for out-of-school girls. Interviews were conducted by one of the authors (MKL), experienced in conducting qualitative interviews and research related to HPV and HPV vaccination.

The interviewer's identity (MKL) as a medical doctor at the highest-level referral hospital in Lusaka could have potentially influenced the mutual relationship with participants and could have influenced how questions were asked. Further, the adolescents could easily feel the power difference and withhold information thinking the interviewer knows it all. The interviewer tried as much as possible to explain her current role as a researcher with a primary role in collecting data. To help the interviewer to be neutral, a reflexivity journal was maintained. Interviews were conducted in the local language, Cinyanja, or English, depending on the participants’ preference. All interviews were audio recorded using a voice recorder and transcribed verbatim.

Participants were allocated unique identification numbers to ensure anonymity during data analysis and discussion of results. All data were anonymized and kept securely. Data were collected until saturation was achieved. Saturation in this case was defined as a point where no additional data was obtained (33). This rigorous data collection process was used to assure dependability.

### Data management and analysis

All interviews were transcribed verbatim by trained research assistants shortly after the interview; each transcript was checked for accuracy by one of the authors by listening to segments of the audio recording. Demographic data were collected to record the characteristics of the girls. Completed and final transcripts were imported into NVIVO 12.0 for data management and analysis.

Thematic analysis was used to identify emerging themes using a six-step approach: (a) familiarisation with the data through close reading of transcripts and memoing, (b) generating initial codes and developing a codebook, (c) searching for themes, (d) reviewing themes, (e) defining and naming themes, and (f) writing up the findings (34). Even though the study was framed within the HBM, thematic analysis was the most ideal approach to capture all aspects of data deductively and inductively. Other studies using the HBM as the guiding framework have used thematic analysis (35).

Transcripts were read thoroughly to understand what the adolescent girls were expressing, and memoing was used to reflect on the data and the analysis process. To assure confirmability, data was checked and rechecked throughout data collection and analysis. The initial codebook was developed (based on the HBM constructs for perceptions deductively) and codes were derived from the memoing process. Coding is an important step in data analysis, as it adds meaning to the data. Initially, one co-author (MKL) coded four transcripts to pilot the codebook, and a second co-author (SF) reviewed the transcripts, any disagreements in coding were discussed until consensus was reached or with a third co-author (MK), this member checking was done to ensure credibility of the data analysis.

The codebook was further refined through this iterative process until it was stable. Health belief model constructs were used as deductive codes. Using the finalised codebook, the rest of the transcripts were coded by MKL. Coded excerpts were then arranged into sub-themes and later themes. Data related to HBM constructs were coded deductively, while other emerging themes were coded inductively (34).

The standards for reporting qualitative research have been followed in the writing of this manuscript (36).

### Ethical considerations

The study was conducted as per the national and international ethical principles in dealing with minors in research. Ethics approval was obtained from the University of Zambia Biomedical Research Ethics Committee (UNZABREC) Ref: 1609-2021, University of the Witwatersrand University Health Research Ethics Committee (HREC Medical) Ref: M21/04/73. A waiver was obtained from the University of North Carolina (UNC) Ethics Committee. Permission was sought from the Ministries of Health and General education and the heads of institutions for the study sites. The study was further registered with the Zambia National Health Research Authority (NHRA). Minors were only allowed to give assent and participate in the study after informed parental consent. Compensation of $5 was given to all participants for their time. The completed assent and parental informed consent forms and interview transcripts were stored separately. Participants were free to withdraw from the study without repercussions, and confidentiality was observed.

## Results

We invited 35 girls to participate in the study. Four girls were ineligible to participate due to lack of parental consent, and one girl was ineligible due to her age (<14 years old, invited by error) and we reached saturation at 30 participants. Of the 30 participants interviewed (age range: 15–18 years old), 17 had received at least one dose of HPV vaccine and 27 were enrolled in school (Table 2). We categorized findings related to the following themes: knowledge about HPV and HPV vaccination, perceived susceptibility to HPV infection, perceived severity of HPV infection, perceived benefits of HPV vaccination, perceived barriers to HPV vaccination, cues to HPV vaccine uptake, self-efficacy, and participants’ suggestions to improve HPV vaccine uptake.

**Table 2:** Participants characteristics.

Participant ID	Sub-district	Age	School	School grade	School type	Vaccinated?
A1	Kanyama	17	Yes	8	Public	No
A2	Matero	15	Yes	9	Public	Yes
A3	Matero	15	Yes	7	Public	Yes
A4	Chelston	18	Yes	12	Public	Yes
A5	Chelston	16	Yes	7	Public	No
A6	Chilenje	15	Yes	11	Private	No
A7	Chelston	15	Yes	9	Quasi	Yes
A8	Chelston	15	Yes	9	Public	No
A9	Kanyama	16	Yes	9	Private	Yes
A10	Chelston	16	Yes	8	Private	Yes
A11	Chelston	16	Yes	7	Public	No
A12	Chawama	16	Yes	8	Private	Yes
A13	Chelston	15	Yes	9	Public	Yes
A14	Kanyama	17	Yes	8	Public	Yes
A15	Chawama	15	Yes	7	Community	Yes
A16	Chawama	15	Yes	7	Community	Yes
A17	Chawama	15	Yes	8	Private	Yes
A18	Chawama	16	Yes	10	Public	No
A19	Chawama	15	Yes	7	Community	Yes
A20	Chilenje	16	Yes	8	Private	No
A21	Chawama	17	Yes	11	Public	No
A22	Chawama	17	Yes	11	Public	Yes
A23	Chawama	16	Yes	10	Public	Yes
A24	Chipata	18	Yes	10	Public	No
A25	Chipata	17	Yes	9	Public	No
A26	Chipata	15	Yes	6	Public	No
A27	Chilenje	16	No	NA	NA	No
A28	Chilenje	16	No	NA	NA	No
A29	Chilenje	15	No	NA	NA	Yes
A30	Chipata	15	Yes	8	Public	Yes

### Knowledge about HPV and HPV vaccine

Participants displayed varying knowledge about HPV and HPV vaccination. Some were knowledgeable about HPV, its consequences, and the role of vaccination. However, some were not aware or knew very little, especially the unvaccinated girls:

*What I know about the HPV vaccine is that it can protect me from having cervical cancer. **A7, vaccinated***

One unvaccinated girl mentioned having easy access to a health facility, but had no information about the HPV vaccine:

*The place to access the vaccine is quite near, but I don’t know anything about the HPV vaccine. **A8, unvaccinated***

While another unvaccinated girl expressed fear due to a lack of awareness:

*We (adolescent girls) are all scared… because we don’t really know what the vaccine is really about. **A27, unvaccinated***

Both vaccinated and unvaccinated participants discussed HPV as a sexually transmitted infection. They also discussed the increased risk of cervical cancer for those who are unvaccinated:

*I know that uh it’s transmitted through sexual intercourse (…) it is found on the foreskin like those men who are not circumcised yet do have sex, and I know that it attacks the cervix of the womb (…) I knew that this is a virus that usually comes from men and I learnt that it is usually on the foreskin of the penis (…) when it comes to a woman it is something some kind like it is foreign, so it reacts. But what I know is that it doesn’t usually react, first takes some time and then it is going to develop into cervical cancer. **A21, unvaccinated***

*This virus is usually found in men at the foreskin, and it is transmitted sexually into the vagina. It won’t react that much the same that day, but it will maybe some years (…) that’s where cervical cancer comes in. **A22, vaccinated***

### Perceived susceptibility

Adolescent girls who reported that they were not sexually active but were thinking about future sexual encounters such as marriage or non-consensual sex such as rape were more conscious about the dangers of HPV and so believed they were more vulnerable to HPV infection. However, some girls felt they were not susceptible to HPV infection as they were not sexually active, hence did not feel the need to take the HPV vaccine until they were older and sexually active. Adolescent girls discussed circumstances beyond their control, such as being raped, which could put them at risk of infection with HPV. Further, there was an inclination towards men being carriers of HPV and not women.

*There are so many ways in which us girls can get the virus (…) like if we get raped, and you (we) can get the virus. So, it is very important that we get the vaccine so that even when I get the devastating news that I have been raped, at least I will not get the HPV. **A14, vaccinated***

*Because if I am not really on the safe side, I can get married to someone who has got that HPV because from what I know is that it is coming from men. So, for example, my husband might have it if I do not have any knowledge about It, and I might end up having cervical cancer. **A21, unvaccinated***

However, when an unvaccinated girl was asked about her risk of being infected with HPV, she said she would not get the virus “because I am not sexually active.” ***A28, unvaccinated***.

While a vaccinated girl had this to say:

*Abstinence is the best way, that’s what I’m practicing so that I can’t get the HPV and maybe others can use condoms if they are married. **A13, vaccinated***

### Perceived severity of HPV infection

Some adolescents reported a higher perception of the severity of HPV infection especially when they were aware of its negative repercussions such as someone getting cervical cancer or experiencing other related health problems including death. Additionally, some perceived that their performance and participation in academic and career-related activities may be affected hence impacting their future professional prospects. Participants reported that their sense of self-worth may suffer as a result of worries about potential health effects and career progression.

*It will affect me as I have a dream of becoming an engineer, but if I have this disease (cervical cancer), I can’t go further in achieving my goals. **A23, vaccinated***

*We were told not to sleep with boys as we would get the virus which doesn’t go away until you die. **A1, unvaccinated***

### Perceived benefits of HPV vaccination

Adolescent girls, regardless of their vaccination status, perceived HPV vaccination as being beneficial. Protection against cervical cancer was the primary benefit that participants discussed. Participants reported that the benefits of the HPV vaccine provided one with a feeling of safety, especially that the vaccine was known to be safe and effective.

*The vaccine is very important to us, it prevents us from getting the cervical cancer. At some point we can be sexually active but if we never had that vaccine, HPV can be easily transmitted into us coz (sic) it comes as a foreign material. So as for that when you get the (HPV) vaccine it will prevent cervical cancer (hmm), it will act as a shield of course. **A22, vaccinated***

*I can say I have not seen any bad side with these uh vaccinations coz (sic) it is just there to prevent those diseases that I might have in future, like cervical cancer, so just better to secure the future. **A21, unvaccinated***

### Perceived barriers to HPV vaccination

There were several barriers reportedly experienced by both vaccinated and unvaccinated. These were coded as subthemes, namely: parental refusal to give consent, belief in myths and misconceptions, negative peer influence, being out of school, and perceived vaccine side effects.

Parents play a key role in the vaccination of their children, as they must consent for them due to their age. Even when adolescent girls may want to get the vaccine, the final decision comes from the parents. Participants reported conflict between mothers and daughters, as some adolescents were willing to receive the vaccine but were discouraged or stopped by parents, especially mothers.

*Some (adolescents) think it’s good, but their parents don’t. So, there may be a bit of conflict between the two owing to the fact that most of my friend’s parents are not educated and hence don’t understand the importance of the vaccine, unlike us students who come here and understand the advantage of getting the vaccine. So, others think it’s good while others don’t. **A13, vaccinated***

*My mum just refused me from getting the vaccine but I really wanted to get the vaccine I heard the effects of this disease (cervical cancer) and really wanted to get the vaccine, but my mum refused. **A20, unvaccinated***

In addition, participants reported that peers also influenced their reactions to the vaccine:

*Well, at my previous school, I was willing to get injected, but after I transferred to this school, I started getting discouraged because of the comments they (peers) used to make such as they are just collecting your blood to take it elsewhere. **A17, vaccinated***

One participant indicated that some adolescent girls choose not to vaccinate because their peers say that one might get other diseases*: “you will be opening the door to other illnesses.” **A27, unvaccinated***.

Belief in myths and misinformation were reported amongst both the vaccinated and unvaccinated girls. These myths seem to be perpetuated by different members of the community (including parents and peers) sharing false information, instilling fear in some girls. The most common myth was that the vaccine is meant to sterilize girls.

*Then there some who say you are not supposed to get the vaccine because you will never have children, you will be barren for the rest of your life. **A7, vaccinated***

*They get wrong information from the community. Some say you may die after taking the vaccine (…) they are told that they have just come to kill us all because there are a lot of females in our population. **A27, unvaccinated***

The misinformation was worsened by the introduction of the COVID-19 vaccine, where the community assumed that adolescents were secretly being inoculated with the COVID-19 vaccine within the pretext of administering the HPV vaccine.

*Others are scared of the injections they think it’s the COVID vaccine. **A2, unvaccinated***

*They think it is expired, harmful, and that it’s the corona vaccine (…) that it’s harmful because they have seen fake information on the internet, that the vaccine can kill, the whites want to kill Africans (…). **A13, vaccinated***

Other myths included the belief that the vaccine can cause illness or death:

*Some were saying that oh maybe these people they are just here to get the blood for this what uh witchcraft activities so they want us to donate our blood. There will be that, they will put us into satanism. **A22, vaccinated***

*I think there may be effects like paralysis of my arm or falling ill, or that I may have a sore at the site of injection that won’t go away and later turn into a cancer (…). They (adolescents) get wrong information from the community. Some say you may die after taking the vaccine… they are told that they have just come to kill us all because there are a lot of females in our population. **A27, unvaccinated***

Another barrier expressed by adolescents was that the vaccination program was biased toward school-going girls:

*They (out-of-school girls) cannot have anyone to go and educate them about the HPV virus that’s the disadvantage for most of them. They are taken into early marriages without consent so that can also prevent them from learning about it (HPV vaccine), and there is no one to educate them. **A13, vaccinated***

### Perceived side effects of HPV vaccine

Some adolescent girls perceived that the HPV vaccine had variable side effects some of which could lead to permanent dysfunction of some body parts which discouraged some from receiving the vaccine.

*It was painful at first then I was uh something was itching so yeah it was very painful at first and then it stopped in three to two days. **A23, vaccinated***

*Then also that you may experience headaches and stomach-aches (…) generally feeling ill. **A8, unvaccinated***

### Cues to HPV vaccination

Participants mentioned getting a recommendation from parents and friends to receive the HPV vaccine. These served an important role as they helped ease and allay fears that eligible adolescents could have.

*I went home to ask my mum if I could get the vaccine, then she said go back! Go and take the vaccine coz (sic) it may protect you somehow as you are growing (…) I was encouraged by my mum to say the vaccine is good that’s how I got motivated. I know since mum is concerned about this let me just do it. **A22, vaccinated***

*My friend is the one who told me to say, next time when they come (vaccinators) you just have to do it, she (a friend) has been encouraging me (…). **A21, unvaccinated***

### Self-efficacy in getting the HPV vaccine

Adolescents showed willingness to take the necessary steps to get the vaccine, and expressed confidence in their own abilities to get vaccinated:

*I think it just with my own motivation coz (sic) I think the health post they are always open (uh hmm) so any day I can just go there (and get the vaccine) yes, coz (sic) even now so I am out of class I have knocked off, I can go and get the vaccine. **A21, unvaccinated***

*I was strongly encouraged and I have that boldness that’s how I got the vaccine. **A22, vaccinated***

### Participants’ suggestions for increasing HPV vaccine uptake

Adolescents had several suggestions for increasing HPV vaccination amidst mixed messages. Some suggestions related to increasing knowledge and awareness of HPV vaccination by making information about HPV vaccination more accessible within communities through social mobilization campaigns, including information on HPV within the school curriculum and active involvement of politicians:

*It should be more like a topic. They should put in a subject like science. Teachers make us understand coz (sic) me I have heard of it and many grade 11s have heard of it. But what of these grade 4s? They only know STIs like HIV. If you ask any child to say what kind of STI do you know, they mention HIV. **A21, unvaccinated***

*Even the vice president needs to say something about the vaccine, encourage the young girls to get the vaccine so that they don’t get diseases such as cervical cancer. **A15, vaccinated***

Some adolescents felt that parents and out-of-school girls should be educated more about the HPV vaccine:

*Well, maybe talking to parents about the benefits of the vaccine, and assure them that nothing bad will happen when their daughters take it. **A15, vaccinated***

*(…) they (Out of school) can be informed by community sensitisation on the advantages of being vaccinated. **A28, unvaccinated***

## Discussion

This study set out to understand adolescent girls’ knowledge and perceptions regarding HPV and the HPV vaccine and discuss its acceptability and uptake implications. The adolescent girls showed variable knowledge of HPV and HPV vaccination as more vaccinated girls were aware of HPV, its transmission route, and the outcome of infection. The attitudes towards the vaccine were generally positive among our study participants. The adolescent girls generally perceived the HPV vaccine as beneficial to limit the spread of HPV and prevent conditions like cervical cancer.

However, several barriers to receiving the HPV vaccine were highlighted, such as lack of parental consent, negative peer influence, belief in myths and misinformation, confusion, and misconceptions around the relationship of the HPV vaccine to the COVID-19 vaccine. Knowledge levels and positive perceptions play an important role in the acceptability of HPV vaccination, however, strategies to overcome the perceived barriers should be identified and implemented to actualize a high uptake (37, 38).

We found that adolescent girls in our study had positive perceptions towards the HPV vaccine, which is likely to increase its acceptability and uptake. Similarly, a qualitative inquiry conducted in Uganda found that girls with good attitudes towards the HPV vaccine widely accepted it despite most of them not having been vaccinated (39). This finding of positive perceptions regarding the HPV vaccine among Zambian adolescent girls holds promise as it could be used to reinforce positive messages about the vaccine to improve future uptake. HPV vaccine uptake remains low in Zambia, for 2021, only 39% and 31% of the eligible girls had received dose one and dose two respectively (16). Therefore, much effort is required to ride on these positive attitudes, because previous studies showed that in some instances, high acceptability did not translate into high HPV vaccine uptake (40, 41).

There was variable knowledge about HPV in general, the route of HPV transmission, and the HPV vaccine itself. Some girls were knowledgeable, while others were not even aware of HPV and its vaccine. In the extant literature, low levels of knowledge (42) have been found in different settings and many times have contributed to low HPV vaccine uptake (43). Similarly, a qualitative study done in the UK early into the HPV vaccination program showed low levels of HPV vaccine knowledge among the recipients (44). Within SSA, a recent study done in Tanzania found low levels of knowledge among adolescents, parents, and teachers, especially before the HPV vaccine was integrated with other health programs (45). Additionally, a quantitative study among Brazilian adolescent girls (46) and a systematic review among European adolescents (47) showed low levels of knowledge.

Therefore, low knowledge of HPV and HPV vaccine is a common feature in different parts of the world and is implicated as one of the contributors to low global HPV vaccination (40). For Zambia, these findings of mixed knowledge among participants in the capital city could be explained by the program being in its infancy, and hopefully, as it matures, more people may be aware through access to information about the HPV vaccine. Therefore, in our study context, culturally appropriate and contextualized strategies could be implemented with the aim of raising awareness and improving knowledge, and subsequently improving HPV vaccine uptake (37).

Examples of strategies include messages around the HPV vaccine packaged in an easy-to-understand format and language using the most accessible platforms: door-to-door campaigns, churches, markets, schools, and health facilities to ensure widespread information sharing. We also suggest that a quantitative inquiry be conducted to measure the actual levels of knowledge using validated tools.

While our participants expressed willingness to recommend the vaccine or intent to get vaccinated, there were some perceived barriers such as lack of parental consent, belief in myths and misconceptions, and negative peer influence. While parental support was viewed as an enabling factor for adolescent girls to receive HPV vaccination, parental refusal to consent was perceived as a significant barrier.

These findings align with other studies showing that parents, specifically mothers, play a key role in influencing the health-related behavior of their daughters (48). When mothers said no, girls did not get the vaccine. In this study, parental refusal superseded the final decision even when girls were willing to receive the vaccine. However, daughters’ parental consent for HPV vaccination in the Zambian context is implied with an opt-out approach, there are no legal requirements for vaccination. Therefore, in a situation where the adolescent insists on receiving the vaccine in the absence of parental consent, the vaccine is given (49).

Our study also showed that when mothers expressed support or encouragement, there was no resistance by the girls. This indicates that mothers would be an essential target group for strategies aiming to increase HPV vaccine uptake in our study setting. Parents and elders in general are held in high esteem, especially in conservative cultures in the African context, and decisions regarding their children are usually final without any further discussion as they are perceived to be in the best interest of the child (50, 51).

Contrary to these findings within the HPV vaccination space, parents in Zambia have actively vaccinated their infants with other childhood vaccines such as oral polio vaccine, Diphtheria, and tetanus vaccines. There has been a gradual increase and sustainable coverage in the period 2000–2018 owing to strong communication, collaboration, and coordination in the background of strong health systems (52). Therefore, considering the relative novelty of the HPV vaccine in Zambia, applying strategies that have been used to improve other vaccine coverage may be necessary (38, 52). Additionally, further studies to understand factors influencing parental decision-making for their daughter's vaccination should be considered to aid in mitigating experienced barriers (17).

There were several myths about the HPV vaccine discussed by adolescent girls in our study, some of which have been reported frequently in the literature. These myths included the vaccine causing infertility (53) and (54) the Western world attempting to eliminate the African population, vaccines being experimental and not safe (44), and the COVID-19 vaccine being given to adolescent girls instead of the HPV vaccine (16). Beliefs in vaccine myths are rampant globally and the HPV vaccine has not been spared (54, 55). Parents who hold fast to myths and misconceptions coupled with low education levels are more likely to decline vaccinating their daughters (56), and as reported in our study, beliefs in myths and misinformation discourage adolescent girls from adopting preventive interventions.

Such findings are worsened by anti-vaccine movements present among society, healthcare workers, and various media platforms misleading many citizens (57). Counter and positive context-specific messages are key in ensuring constant debunking of these myths and misconceptions such as the HPV vaccine does not cause ovarian insufficiency as early claims were based on case reports (58). Dispelling myths and misinformation is, however, not easy, and health education alone is not sufficient, implementing multiple context specific strategies is of paramount importance (37).

In our study, peers were reported to have mixed roles to encourage or discourage vaccination most probably due to the variations in levels of knowledge. These findings on the role of peer influence are not unique to our study but have been elicited elsewhere (59, 60). Adolescence is a period where there is a lot of succumbing to “peer pressure” as adolescents seek a sense of belonging and validation by peers. In some instances, school vaccination programs have led to mass psychogenic effects and this has negatively affected the vaccination programs (61). Therefore, with active engagement and education, peers can play a critical role in increasing HPV vaccine uptake if they are knowledgeable and they themselves have experienced vaccination.

Some of our participants had a low perceived risk and susceptibility to HPV infection, which appears to be a common finding for people who are not sexually active (35). The asymptomatic nature of early HPV infection and marketing of the vaccine as a prevention for sexually transmitted disease in contrast with other childhood vaccines could contribute to this perception. A study in Ghana on risk perception for developing cervical cancer also showed lower risk perception compared to the actual risk (62). Therefore, ongoing education for adolescent girls and parents on HPV virus infection disease progression is of paramount importance. These key stakeholders must be educated on the importance of the HPV vaccine being given before they become sexually active.

### Strengths and limitations

This study has some strengths in that the voices of the primary HPV vaccine recipients have been illuminated. Out-of-school adolescents and those in hard-to-reach areas are rarely included in studies. We included out-of-school girls hence increasing the transferability of our findings in situations with in-school and out-of-school girls.

The use of the HBM as a guiding framework could be a basis for future quantitative research among adolescent girls with a view to ascertain whether some of the perceptions and experienced barriers are associated with HPV vaccine uptake and how they could be mitigated. Further, even though the current national guidelines entail a girls-only vaccination program, a future study involving boys is necessary because as the vaccine becomes more available, gender-neutral HPV vaccination will become policy.

To the best of our knowledge, this is the first study exploring knowledge, perceptions, and suggestions to increase the HPV vaccine from the perspective of adolescent girls in Zambia since the launch of the national program in 2019. It reinforces the earlier findings by other researchers among multiple stakeholders of low to moderate knowledge, positive attitudes, and perceived barriers amid high acceptability and low HPV vaccine uptake. It is important for policymakers to consider suggestions made by adolescent girls for the continued program implementation.

Our study is not without limitations. This study is qualitative and the participants were purposively sampled, therefore our results may vary with other settings if we randomly sampled a higher number of adolescent girls. This study enrolled participants in a single setting (Lusaka, Zambia), further investigation may be needed to confirm whether findings are generalizable to other settings, hence may affect transferability. These findings are however, still relevant within the Zambian context, and could still be useful in other sub-Saharan African countries.

Further, this study did not ask the girls what their HIV status was, therefore there is no information that is specifically about HIV keeping in mind the increased risk of HPV acquisition and cervical cancer development among people living with HIV.

We do not report differences in health beliefs between sub-groups as our goal was not to achieve saturation in the subgroups of vaccinated and unvaccinated, as well as in-school and out-of-school girls. We focused on the emerging themes from the whole data set other than the disaggregated data as girls were not sampled in that way. Additionally, there were only three out-of-school girls.

To address these limitations, we have included a thorough explanation of the sampling methods, discussed our findings within the context of existing literature, supported findings with quotes from our interviews reached saturation within our sample, and engaged in an iterative data collection and analysis process, thus increasing the dependability and transferability of findings.

## Conclusion

For the first time since the nationwide roll out of the HPV vaccination program in Zambia we have highlighted the knowledge and perceptions of adolescent girls regarding the HPV vaccine, illuminating factors that may influence HPV vaccine acceptability and uptake in Zambia, from the perspective of adolescent girls. Our findings on adolescent perceptions are similar to those other earlier studies in Zambia have found with different stakeholders.

The knowledge that the HPV vaccine can provide long-term protection may improve uptake. There are still urgent issues that need to be addressed such as myths and misinformation, and lack of parental consent to vaccinate daughters. To promote greater knowledge and boost vaccination uptake, ongoing efforts must be made to offer correct information, address misinformation, and improve understanding by adolescents and parents on HPV infection and the advantages of HPV vaccination.

Additionally, we recommend that future studies enroll adolescent girls based on school status and to reach saturation considering that out-of-school girls are difficult to just as has been shown in this study, despite their high vulnerability to HPV infection and its consequences.

## Data Availability

The original contributions presented in the study are included in the article/Supplementary Material, further inquiries can be directed to the corresponding author.
